# *Streptococcus pneumoniae* Proteins AmiA, AliA, and AliB Bind Peptides Found in Ribosomal Proteins of Other Bacterial Species

**DOI:** 10.3389/fmicb.2017.02688

**Published:** 2018-01-15

**Authors:** Fauzy Nasher, Manfred Heller, Lucy J. Hathaway

**Affiliations:** ^1^Institute for Infectious Diseases, Faculty of Medicine, University of Bern, Bern, Switzerland; ^2^Graduate School for Cellular and Biomedical Sciences, University of Bern, Bern, Switzerland; ^3^Department of Clinical Research, Proteomics and Mass Spectrometry Core Facility, University of Bern, Bern, Switzerland

**Keywords:** *Streptococcus pneumoniae*, peptide, ABC transporter, Ami–AliA/AliB permease, interspecies communication

## Abstract

The nasopharynx is frequently colonized by both commensal and pathogenic bacteria including *Streptococcus pneumoniae* (pneumococcus). Pneumococcus is an important pathogen responsible for bacterial meningitis and community acquired pneumonia but is also commonly an asymptomatic colonizer of the nasopharynx. Understanding interactions between microbes may provide insights into pathogenesis. Here, we investigated the ability of the three oligopeptide-binding proteins AmiA, AliA, and AliB of an ATP-binding cassette transporter of pneumococcus to detect short peptides found in other bacterial species. We found three possible peptide ligands for AmiA and four each for AliA and AliB of which two for each protein matched ribosomal proteins of other bacterial species. Using synthetic peptides we confirmed the following binding: AmiA binds peptide AKTIKITQTR, matching 50S ribosomal subunit protein L30, AliA binds peptide FNEMQPIVDRQ, matching 30S ribosomal protein S20, and AliB binds peptide AIQSEKARKHN, matching 30S ribosomal protein S20, without excluding the possibility of binding of the other peptides. These Ami–AliA/AliB peptide ligands are found in multiple species in the class of Gammaproteobacteria which includes common colonizers of the nostrils and nasopharynx. Binding such peptides may enable pneumococcus to detect and respond to neighboring species in its environment and is a potential mechanism for interspecies communication and environmental surveillance.

## Introduction

*Streptococcus pneumoniae* (pneumococcus) continues to be an important human pathogen, responsible for high global morbidity and mortality despite the availability of antibiotics and the current 13-valent polysaccharide conjugate vaccine. Pneumococcus is commonly a colonizer in the nasopharynx and it shares the niche with many other microorganisms. Pneumococcus has more than 60 ABC transporters, with many conserved between strains (Durmort and Brown, 2015). ABC transporters play a role in widespread cellular functions particularly import and export of substrates including micronutrients, amino acids, peptides, sugars, antibiotics, and antimicrobial peptides (Durmort and Brown, 2015). The first ABC transporter to be discovered in Gram-positive bacteria is encoded by the Ami locus and is homologous to the oligopeptide permease (Opp) of *Salmonella typhimurium* and *Escherichia coli* (Alloing et al., 1990). The transporter, named Ami-AliA/AliB permease, is composed of three substrate binding lipoproteins AmiA, AliA and AliB, AmiC, and AmiD which make up the transmembrane domains of the transporter and AmiE and AmiF which are the ATPases (Alloing et al., 1990, 1994). Genes encoding *amiA* and *amiC* to *amiF* are part of the same operon while *aliA* and *aliB* are found elsewhere in the pneumococcal genome (Alloing et al., 1990). Ami-AliA/AliB has been suggested to play a role in environmental signaling, triggering competence (Claverys et al., 2000) as well as having a role in successful colonization of the nasopharynx (Kerr et al., 2004). Using *amiA, aliA*, and *aliB* mutants, Alloing et al. (1994) concluded that the lipoproteins had overlapping specificities and were responsible for the uptake of various oligopeptides particularly those containing leucine and arginine. This conclusion was reached by observing pneumococcal growth rather than looking directly for evidence of binding of the lipoproteins to oligopeptides. Previously we found homologs of *aliB* in nonencapsulated pneumococcus in place of capsule genes and named them *aliB*-like ORF1 and ORF2 (Hathaway et al., 2004). These genes are widespread globally in the nontypeable pneumococcal population (Hilty et al., 2014). AliB-like ORF1 and 2 are able to sense short peptide fragments found in other bacterial species in the nasopharynx (Hathaway et al., 2014). However, as nonencapsulated pneumococci are generally avirulent, here we looked whether a similar mechanism might occur in virulent pneumococcus. We identified and confirmed binding of peptide ligands for AmiA, AliA, and AliB and found that their sequences matched ribosomal proteins of other bacterial species.

## Materials and Methods

### Bacterial Culture

Bacteria, stored at -80°C using Protect bacterial preservers (Technical Service Consultants, Heywood, United Kingdom), were grown on Columbia sheep blood agar (CSBA) plates at 37°C, 5% CO_2_. Overnight cultures were prepared with 5–10 colonies in 5 ml brain heart infusion (BHI) broth (Becton Dickinson and Company, Le Pont-de-Claix, France) containing 5% fetal calf serum (FCS) (Biochrom KG, Berlin, Germany).

### Protein Expression

Proteins AmiA, AliA, and AliB were expressed in *E. coli* BL21 strain (Novagen, Darmstadt, Germany) and purified using an N-terminal glutathione *S*-transferase (GST)-tag. Swiss nonencapsulated nasopharyngeal pneumococcal isolate 110.58 (accession number CP007593) MLST 344 (Hathaway et al., 2004) DNA was used as template for PCR using the following primers as a template; for *amiA*, forward primer 5′-TTTCCCGGGCGGCCCCTAAAGCTTATGGCTATGTTTATACAGC-3′ and reverse primer 5′-AAACTCGAGTTAATGATGATGATGATGATGACGTTACTTCACATGACTTGCCAATTCTTTT-3′; for *aliA*, forward primer 5′-TTTCCCGGGCGCAAGGTGAAAAGACCTTTGCGTTTACCTACGA-3′ and reverse primer 5′-AAACTCGAGTTAATGATGATGATGATGATGACGTTATTTCACATGTTTTGCAAGTCTTCT-3′; for *aliB*, forward primer 5′-TTTCCCGGGCGAAAACATACAACTATGTTTATTCAAGTGATCC-3′ and reverse primer 5′-AAACTCGAGTTAATGATGATGATGATGATGACGTTATTTGACATGTTTTGCCAATTCTT-3′. Primers were used to amplify the sequence encoding the full-length protein excluding the 5′-portion encoding the predicted N-terminal signal peptide (predicted using Center for Biological Sequence Analysis – SignalP 4.1 server^1^). Underlined are the restriction enzyme sites for *XmaI* (forward primer) and *XhoI* (reverse primer) to enable insertion into the vector. The amplification products were ligated into expression plasmid pGEX 6p-1 which has an ampicillin resistance cassette. The construct was used to transform the *E. coli* which harbors a “codon plasmid” adapted for Gram-positive bacteria which contains a chloramphenicol selection marker.

Expression of GST-*amiA*-His_6_ and GST-*aliA*-His_6_ was induced by isopropyl-β-D-thiogalacto-pyranoside (IPTG), final concentration 0.1 mM, shaking overnight at 20°C. For GST-*aliB*-His_6_, expression was induced by IPTG at a final concertation of 1 mM with shaking at 37°C for 4 h. Protein was isolated by binding the GST-tag to GSH-agarose beads (Thermo Fisher Scientific, Bremen, Germany). The recombinant proteins were analyzed by SDS–PAGE (Supplementary Figure S1) and the identities were confirmed by nano LC/MS for unambiguous protein identification.

### Identification of Peptide Ligands

Ligand binding was investigated based on the method described previously (Hathaway et al., 2014). The recombinant proteins were incubated with pooled nasal swabs collected in Universal Transport Medium (Copan, Italia) from an anonymous healthy child during the first year of life, at 37°C on a turning wheel for 1 h to allow binding. Nasal swabs were kindly provided by Professor Philipp Latzin (University of Bern Children’s Hospital, Inselspital, Bern, Switzerland) in the context of a larger study (Korten et al., 2016, 2017) and stored at -80°C until use. To determine whether the source of any peptides discovered was the nasal swabs, the recombinant proteins were also incubated with PBS, instead of nasal swabs, as a control.

The proteins with ligands bound were then recovered as described above using GSH-agarose beads. Bound ligands were released by denaturing the proteins with 8 M urea. As a negative control, the same procedure was performed in parallel with the exception that no IPTG was added to the bacteria. Therefore, specific induction of protein was avoided in the negative control, leaving only expression of the *E. coli* proteins. Released peptides were isolated using 30 KDa filters then analyzed by LC–MS/MS.

The peptides were sequenced on a QExactive HF mass spectrometer coupled with an Easy-nLC 1000 (Thermo Fisher, Bremen, Germany) with one injection of 5 μl eluate. Peptides were trapped on a pre-column [C18 PepMap100, 5 μm, 100 Å, 300 μm × 5 mm (ThermoFisher Scientific, Reinach, Switzerland)] and separated by backflush on a C18 column (C18, 3 μm, 155 Å, 0.075 mm i.d. × 150 mm length, Nikkyo Technos, Tokyo, Japan) by applying a 40 min gradient of 5% acetonitrile to 40% in water, 0.1% (V/V) formic acid, at a flow rate of 350 nl/min^-1^.

Data acquisition was in data-dependent mode on the top 15 peaks with mass-to-charge ratio (*m*/*z*) between 360 and 1600, charge state 2–7, and no exclusion time set. Survey full scan MS resolution was set at 60,000 (at 400 *m/z*), automatic gain control (AGC) target of 1e6 with a maximum fill time of 50 ms. Higher energy collisional dissociation (HCD) fragmentation was carried out with a normalized collision energy of 27%, AGC of 1e5, maximum injection time of 110 ms, and spectra were recorded with a resolution of 15,000. Mascot generic files (mgf) were created with Proteome Discoverer 2.1 (ThermoFisher Scientific, Reinach, Switzerland) from the ^∗^.RAW files by combining to one spectrum entry all fragment spectra from the same precursor mass with an allowed delta mass of 10 ppm and a maximum retention time shift of 3 min. Fragment spectra interpretation was done with Easyprot using the following search parameters: parent error tolerance 10 ppm, C-terminal cleavage after all amino acids except proline with 20 missed cleavages and one non-specific cleavage site allowed, dynamic amino acid modifications of one oxidation on Met and one protein N-terminus acetylation, minimal peptide *z*-score of 5, max *p*-value 0.01, searching against all bacteria protein sequences present in the Uniprot-tremble database from May 2015. The apparent false peptide identification rate was approximately 30% as estimated by a parallel search against the reversed database. The discovery of peptides specifically bound to AmiA, AliA, and AliB was achieved by comparison of peak features of the negative with the positive control using the software SIEVE version 2.1 (ThermoFisher Scientific, Reinach, Switzerland).

Peptide candidate spectra interpretations by Easyprot were manually validated and possible sources of the peptide were identified by performing BLAST searches using the NCBI website^2^.

### Tryptophan-Binding Assay

Intrinsic tryptophan fluorescence spectroscopy assay was performed to determine binding of synthesized peptides (PolyPeptide Group, Strasbourg, France) to the recombinant proteins based on methods described previously (Thomas et al., 2006; Basavanna et al., 2009). Amicon Ultra centrifugal filters (Millipore, Carrigtwohill, Ireland) were used to exchange the elution buffer for the binding assay buffer. The assay mixture consisted of 0.5 μM GST-*amiA*-His_6_ or GST-*aliA*-His_6_ or GST-*aliB*-His_6_ in 1.5 ml 50 mM Tris–HCl pH 8.0, to which peptides dissolved in 10 mM NaH_2_PO_4_ were added to a final concentration of 250 μM. The mixture was continuously stirred at 25°C. Fluorescence was measured by LS-5B luminescence spectrometer (PerkinElmer, Schwerzenbach, Switzerland), connected to a graph printer, at excitation wavelength 295 nm (slit width 5 nm) and emission wavelength 330 nm (slit width 10 nm).

## Results

**FIGURE 1:**
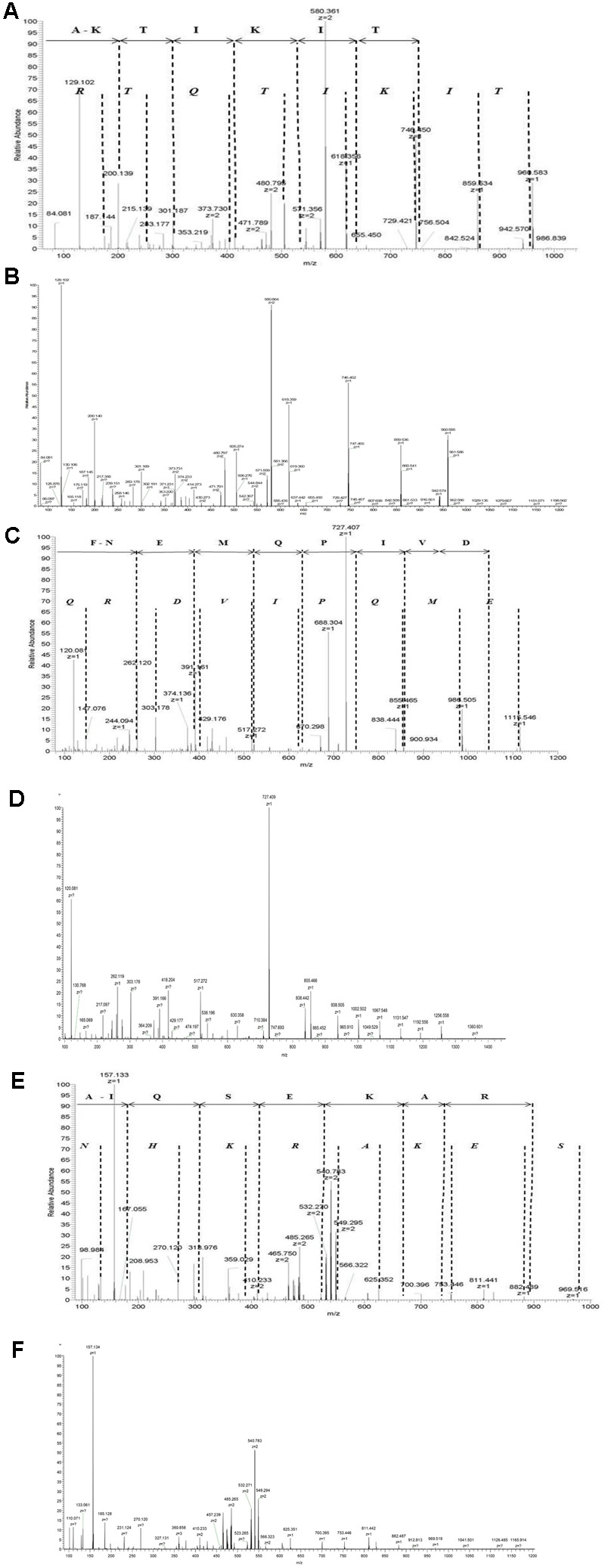
Identification of AmiA peptide ligand AKTIKITQTR, AliA peptide ligand FNEMQPIVDRQ, and AliB peptide ligand AIQSEKARKHN. Manual interpretation of the fragment peaks is shown using non-italic letters for the *b-*ion (fragments appearing to extend from the N-terminal fragment) and italic letters for the *y*-ion. Representative fragment mass spectrum was confirmed for correct interpretation with fragment spectra of synthetic peptides **(B, D, F)**. **(A)** AmiA peptide ligand: extracted ion chromatogram of the doubly charged peptide ion with mass-to-charge ratio (*m*/*z*) of 580.361 [retention time (RT) 15.98 min]. The *b*-ion series (fragments appearing to extend from the N-terminal) is annotated. Individual peaks for the last three amino acids Q, T, and R at the amino terminus are not visible in the *b*-ion series and are therefore not annotated, but Q–T–R is the mass difference between the last detectable *b*-ion at 756.504 and the molecular ion of 1141.70. **(C)** AliA peptide ligand: extracted ion chromatogram of the single charged peptide ions with mass-to-charge ratio (*m*/*z*) of 688. 83 (RT = 12.85 min, corresponding to sequence FNEMQPIVDRQ). Peaks corresponding to the two amino acids at the c-terminal, R and Q are not visible in the *b*-ion series and are therefore not annotated; R–Q is the mass difference between the last detectable *b*-ion at 1074.49 and the molecular ion of 1358.65. **(E)** AliB ligand: extracted ion chromatogram of the doubly charged peptide ions with mass-to-charge ratio (*m*/*z*) of 427.906 (RT = 12.85 min, corresponding to sequence AIQSEKARKHN). Individual peaks corresponding to the last three amino acids K, H, and N at the c-terminal are not visible in the *b*-ion series and are therefore not annotated, but K–H–N is the mass difference between the last detectable *b*-ion at 884.49 and the molecular ion of 1263.69. Manual interpretation of fragment peak for other peptides we identified can be found in Supplementary Figure S2.

We expressed recombinant proteins AmiA, AliA, and AliB and incubated them in pooled resuspended nasal swabs from a healthy child to capture specifically bound ligands. Bound peptide ligands were then released by denaturing the proteins and identified by LC-MS. Manual *de novo* peptide sequencing aided by a standard database match approach was performed to confirm the sequences of the peptides from their MS/MS spectra (**Figure 1** and Supplementary Figure S2).

We found a total of 11 possible peptide ligands; three for AmiA and four each for AliA and AliB (**Table 1**). BLAST analysis indicated the following specific matches to proteins: AmiA ligands AKTIKITQR and LQEHSVILIRG are part of ribosomal proteins of multispecies in the class of Gammaproteobacteria, including *Moraxella* spp., *Klebsiella* spp., *Haemophilus* spp., and *Pasteurella* spp., while SVVNDTDGIVRVAE is a part of galactitol-1-phosphate 5-dehydrogenase protein from *E. coli*. The sequences for AliA peptide ligands FNEMQPIVDRQ and MTADAVKQVEEMLA are part of ribosomal proteins of multispecies in the class of Gammaproteobacteria, Firmicutes, and Actinobacteria, including *Bacillus* spp., *Microbacteria* spp., *Clostridium* spp., and *Corynebacterium* spp., while VEELKPTEP and VPVTVPLM are part of hypothetical protein of *Streptomyces prinstinaespiralis* and hydroxylamine reductase of *Sarcina* species, respectively. AliB peptide ligands AIQSEKARKHN and VMVKGPGPGREST are ribosomal protein of multispecies in the class of Gammaproteobacteria. PIVGGHEGAGV and TFKEKVM are part of hypothetical protein of *Streptomyces* spp. and hydroxylamine reductase of *Sarcina* spp., respectively. We then repeated the protein expression and ligand binding and found the same three peptides for AmiA, the same four peptides for AliB but failed to detect any peptide ligands for AliA on the second attempt. We noted that for each protein, two of the peptides matched sequences found in ribosomal proteins of other bacterial species. These six peptides were synthetically synthesized but only three were soluble and were therefore tested in the binding assay. These three peptides are highlighted in bold in **Table 1**.

**Table 1:** AmiA, AliA, and AliB oligopeptide-binding proteins and their putative ligands.

Protein	Peptide sequences	Possible origin determined by BLAST analysis
**AmiA**	**AKTIKITQTR**	**50S ribosomal protein L30 (multispecies)**
	LQEHSVILIRG	30S ribosomal protein S12 (multispecies)
	SVVNDTDGIVRVAE	Galactitol-1-phosphate 5-dehydrogenase (*Escherichia coli*)
**AliA**	**FNEMQPIVDRQ**	**30S ribosomal protein S20 (multispecies)**
	MTADAVKQVEEMLA	50S ribosomal protein L4 (multispecies)
	VEELKPTEP	Hypothetical protein (*Streptomyces* spp.)
	VPVTVPLM	Hydroxylamine reductase (*Sarcina* spp.)
**AliB**	**AIQSEKARKHN**	**30S ribosomal protein S20 (multispecies)**
	VMVKGPGPGREST	30S ribosomal protein S11 (multispecies)
	PIVGGHEGAGV	NDMA-dependent alcohol dehydrogenase (multispecies)
	TFKEKVM	Phospho-sugar mutase (multispecies)

To determine the source of the peptides we also analyzed the peptides recovered from the recombinant proteins incubated with PBS instead of nasal swabs. We found that all three AmiA peptides could be detected as well as AliA peptide VPVTVPLM and AliB peptides VMVKGPGPGREST and PIVGGHEGAGV but not the AliA peptides FNEMQPIVDRQ, MTADAVKQVEEMLA, or VEELKPTEP, or the AliB peptide AIQSEKARKHN. Since the recombinant proteins are expressed in *E. coli* and several of the peptides are found in *E. coli*, it appears that some binding occurs during protein synthesis. The *E. coli* are cultured in Luria broth which contains both tryptone and yeast extract which may also be sources of peptides for the recombinant proteins which have not been exposed to the nasal swabs.

So, of the three peptides matching ribosomal proteins that were tested in the binding assays, the ligands of AliA and AliB were found only after exposure to nasal swabs but AmiA appeared to bind its ligand during protein synthesis in *E. coli*.

**FIGURE 2:**
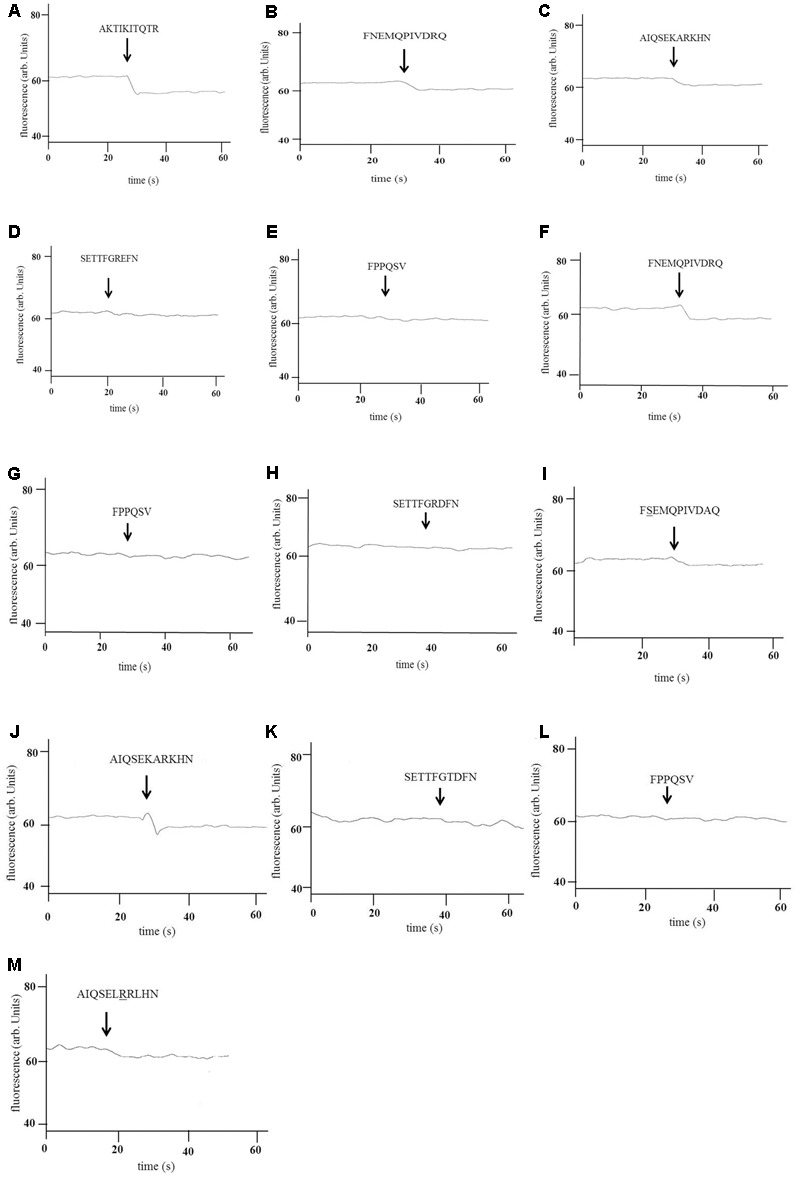
Binding of AmiA, AliA, and AliB to their peptide ligands. Binding of recombinant AmiA **(A–E)**, AliA **(F–I)**, and AliB **(J–M)** to the peptides shown was determined by intrinsic tryptophan fluorescence-binding assay. A drop in fluorescence indicates binding. Peptide sequences are indicated above the arrows marking the point of their addition to the protein. A representative of three independent experiments, performed on different days, is shown. AmiA bound its predicted ligand **(A)** but also the ligands of AliA **(B)** and AliB **(C)** but not the ligands of AliB-like ORF 1 **(D)** and AliB-like ORF 2 **(E)** which acted as negative controls. AliA also bound its predicted ligand **(F)** but not the negative controls **(G,H)**. There was also a drop in fluorescence upon exposure to its ligand with one amino acid change (underlined) **(I)** although this was less evident than in **(F)**. AliB also bound its predicted ligand **(J)** but neither of the negative controls **(K,L)** or to a great extent to its ligand with one amino acid change (underlined) **(M)**.

Tryptophan fluorescence binding assays confirmed that AmiA-bound AKTIKITQTR (**Figure 2A**) and also to some extent to AliA and AliB putative peptide ligands (**Figures 2B,C**), but not the ligands of AliB-like ORF 1 and ORF 2 which acted as negative controls (**Figures 2D,E**). Tryptophan fluorescence assay also confirmed AliA peptide ligand to be FNEMQPIVDRQ (**Figure 2F**). AliA protein did not bind AmiA or AliB peptide ligands (data not shown) or the negative control peptides (**Figures 2G,H**). AliB peptide ligand was confirmed to be AIQSEKARKHN (**Figure 2J**), AliB did not bind the peptide ligands of AmiA and AliA (data not shown) or the negative control peptides (**Figures 2K,L**). To check the specificity of the binding of the proteins to their ligands, we used BLAST to identify sequences of the peptide ligands with a naturally occurring change of an amino acid (underlined). The peptides were synthesized and binding was tested; AQTIKITQTR, matching 50S ribosomal protein L30 of multispecies, was insoluble. FSEMQPIVDRQ and AIQSEKRRKHN, matching 30S ribosomal protein S20 of multispecies bacteria in the class of Gammaproteobacteria, including *Klebsiella* species, were soluble and were used in the tryptophan-binding assay: There was a drop in fluorescence when AliA was exposed to FSEMQPIVDRQ (**Figure 2I**) although to a lesser extent than to its unmodified ligand (**Figure 2F**). AliB did not show binding to any great extent to its modified ligand AIQSEKRRKHN (**Figure 2M**).

## Discussion

The pneumococcal ABC transporter Ami-AliA/AliB has been suggested to have a role in the uptake of peptides to detect the environment (Claverys et al., 2000) and we have previously shown two homologs of AliB in nonencapsulated *S. pneumoniae* bind specifically peptides matching ribosomal proteins of other species, indicating a potential route for interspecies communication (Hathaway et al., 2014).

Here, our aim was to determine whether the oligopeptide-binding proteins of the Ami-AliA/AliB transporter of *S. pneumoniae* are able to bind peptides matching other bacterial species. We cloned and expressed recombinant AmiA, AliA, and AliB, incubated the proteins with resuspended nasal swabs pooled from a healthy child, and identified their peptide ligands. We found peptides matching sequences of ribosomal proteins of other bacterial species that are members of the phyla Proteobacteria, Firmicutes, and Actinobacteria, common colonizers of the nostrils and nasopharynx in prepubertal children (Pettigrew et al., 2012; Biesbroek et al., 2014; Mika et al., 2015; Teo et al., 2015). Although FNEMQPIVDRQ (AliA ligand) and AIQSEKARKHN (AliB ligand) were only bound to their respective proteins following exposure to nasal swabs, AKTIKITQTR (AmiA ligand) was already bound to AmiA before exposure to nasal swabs indicating binding during recombinant protein synthesis in the *E. coli*. However, during natural expression in *S. pneumoniae* we would expect all three proteins to be produced without their ligands bound since none of the ligands are present in the *S. pneumoniae* bacteria themselves. Bacteria have been known to secrete proteins extracellularly despite the absence of a signaling peptide (Bendtsen et al., 2005) and ribosomal proteins are abundant and have been isolated extracellularly, and are often found on the surface of bacteria cells (Jeffery, 2003). Lee et al. (2007) also showed that the secreted proteins of *E. coli* included ribosomal proteins. It would not be improbable that the pneumococcus has evolved to detect and respond to its neighbors through short peptide fragments of the ribosomal proteins, since ribosomal proteins are highly conserved (Ban et al., 2014).

We have shown that AmiA is able to bind to AliA and AliB peptide ligands as well as its own, which is in line with the results of Claverys et al. (2000) whose investigation of different combinations of *amiA, aliA*, and *aliB* mutants indicated that the proteins may have overlapping specificity to oligopeptides. Redundancy in the Ami-AliA/AliB oligopeptide-binding proteins has also been reported in a mouse model of nasopharynx colonization where AmiA and AliA could compensate for the absence of AliB (Kerr et al., 2004). AliA and AliB are homologs of AmiA with over 60% amino acid identity; this could explain why AmiA is able to bind peptide ligands of AliA and AliB. Our findings show that the overlapping specificity to oligopeptides is at the level of binding and so the specificity of signaling could be determined intracellularly.

Oligopeptide-binding proteins have been shown to transport intercellular signaling peptides in Gram-positive bacteria *Lactococcus lactis* (Lamarque et al., 2004), *Bacillus subtilis* (Perego and Hoch, 1996; Jiang et al., 2000), and Gram-negative bacteria *E. coli* and *S. typhimurium*, ranging in size from 3 to 20 amino acids (Guyer et al., 1986). Signaling peptides have been identified to play a role in genetic competence in *S. pneumoniae* (Pearce et al., 1994; Alloing et al., 1996; Hathaway et al., 2014), extracellular virulence factor production in Group A streptococci (Darmstadt et al., 2000), production of adhesins in *Streptococcus gordonii* (McNab and Jenkinson, 1998) and regulation of plasmid transfer in *Enterococcus faecalis* (Leonard et al., 1996). It is likely that pneumococcus transports extracellular peptides from other species to regulate a diverse range of processes in the cell. In a future study, it would be interesting to investigate how AmiA, AliA, and AliB peptide ligands affect the pneumococcal phenotype.

We propose that binding of short peptide fragments of neighboring species in the microbiota could be a mechanism for communication among species. This could be a target for intervention strategies and potential vaccine candidates, in the current era where pneumococcal disease is still an enormous global burden especially in young children and the elderly.

## Author Contributions

LH conceived the study. FN drafted the manuscript. FN and LH participated in designing the study. FN performed cloning and expression of recombinant protein, *de novo* sequencing, and tryptophan-binding assay. MH confirmed purified recombinant protein, identified peptide ligands, and verified *de novo* peptide sequencing. All authors were involved in data interpretation and gave final approval for publication.

## Conflict of Interest Statement

The authors declare that the research was conducted in the absence of any commercial or financial relationships that could be construed as a potential conflict of interest.

## Footnotes

1 http://www.cbs.dtu.dk/services/SignalP/

2 http://www.ncbi.nlm.nih.gov/BLAST/
