# Supplementary material for: Streptococcus pneumoniae Proteins AmiA, AliA, and AliB Bind Peptides Found in Ribosomal Proteins of Other Bacterial Species
Source: Front Microbiol. 2018 Jan 15;8:2688. doi: 10.3389/fmicb.2017.02688 (PMC5775242; doi:10.3389/fmicb.2017.02688)
Supplement: Supplementary file 1 [file Image_1.PDF]

Supplementary Material

Streptococcus pneumoniae proteins AmiA, AliA and AliB bind peptides found in ribosomal proteins of other bacterial species

Fauzy Nasher, Manfred Heller, Lucy J. Hathaway

*** Correspondence:** lucy.hathaway@ifik.unibe.ch

Supplementary Figure S1

A)

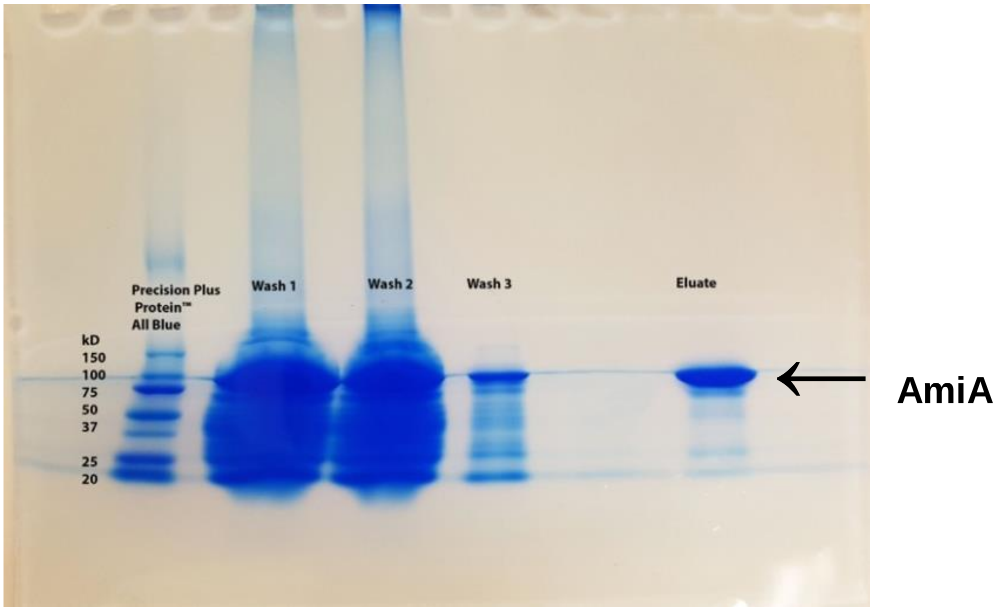

B)

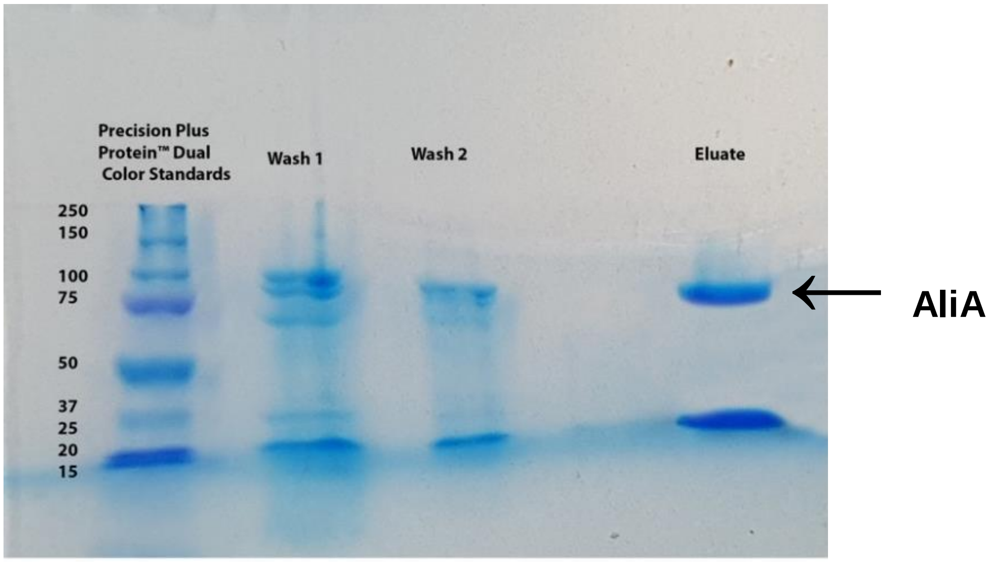

C)

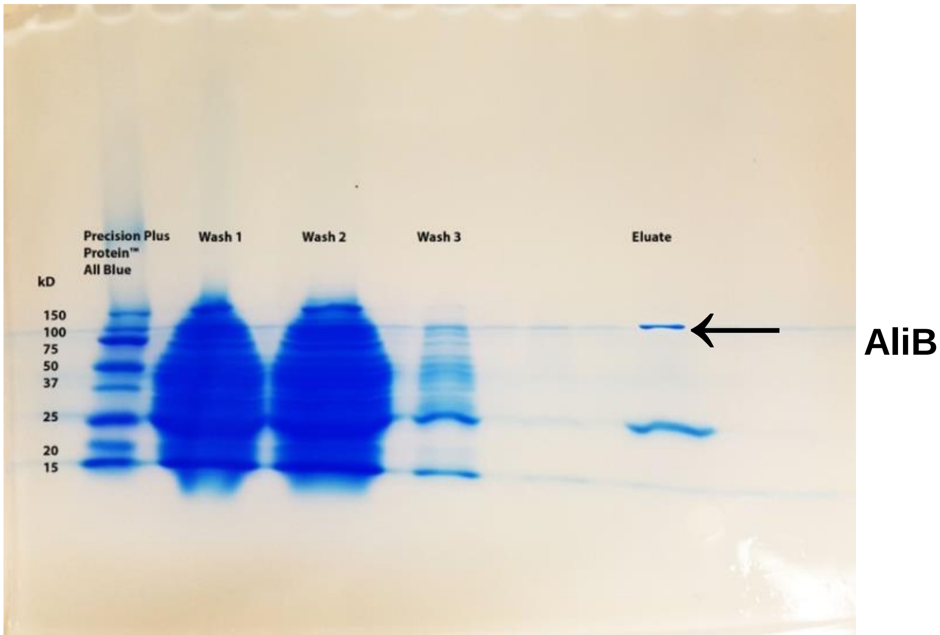

Figure S1. SDS-PAGE of proteins AmiA, AliA and AliB before and after purification. To express the proteins the bacteria were streaked out on LB plates containing 100 µg/ml ampicillin and incubated overnight at 37°C and the colonies used to inoculate LB broth containing 100 µg/ml ampicillin. After growth at 37°C with shaking until OD_{600nm} = 0.5 for AmiA and AliA expression was induced by adding IPTG to 0.1mM and incubated overnight at 20 ^{o}C, for AliB culture was grown with shaking until OD600nm = 1, expression was induced by adding IPTG to 1mM followed by incubation at 37 ^{o}C for 4 hours. The bacteria were recovered by centrifugation and the pellet resuspended in lysis buffer (50 mM NaH_2PO_4,300 mM NaCl,10 mM imidazole, pH adjusted to 8 using NaOH). Lysozyme was added to give 1 mg/ml, followed by incubation on ice for 30 minutes and then sonication 6 times for 10 seconds with 10 seconds cooling period between each sonication, maintaining on ice during sonication. The supernatant containing the soluble protein was recovered by centrifugation 4°C, 10 000g for 20 min. Purification of protein was by its N-terminal GST tag as described in the main text.

Supplementary Figure S2

(A)

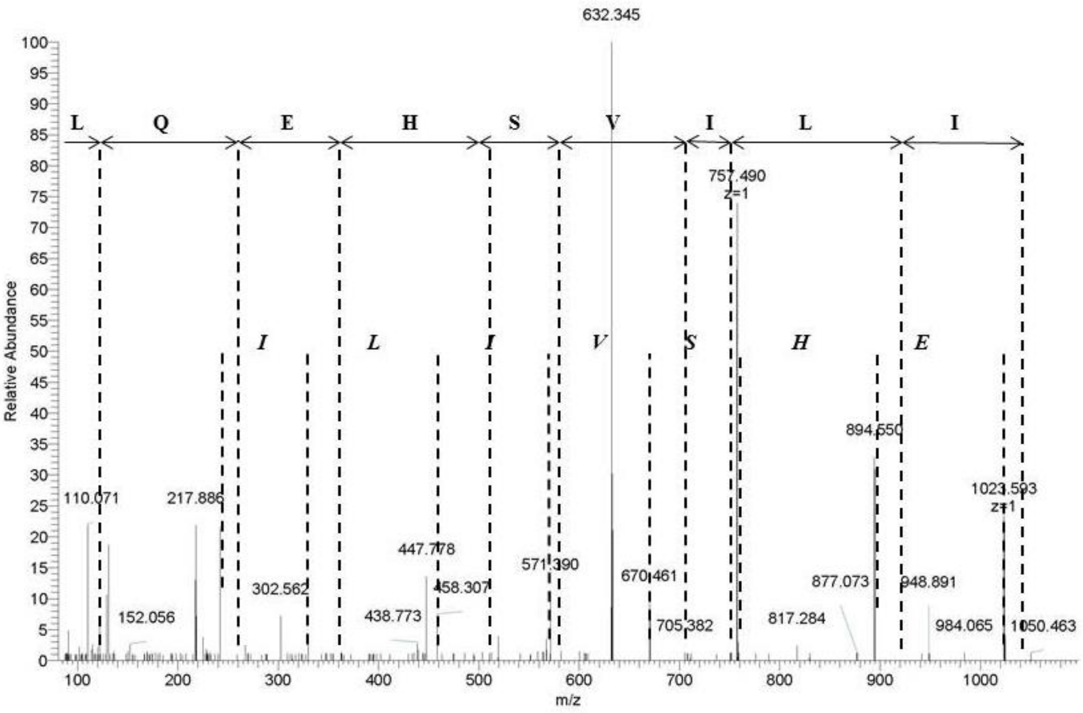

(B)

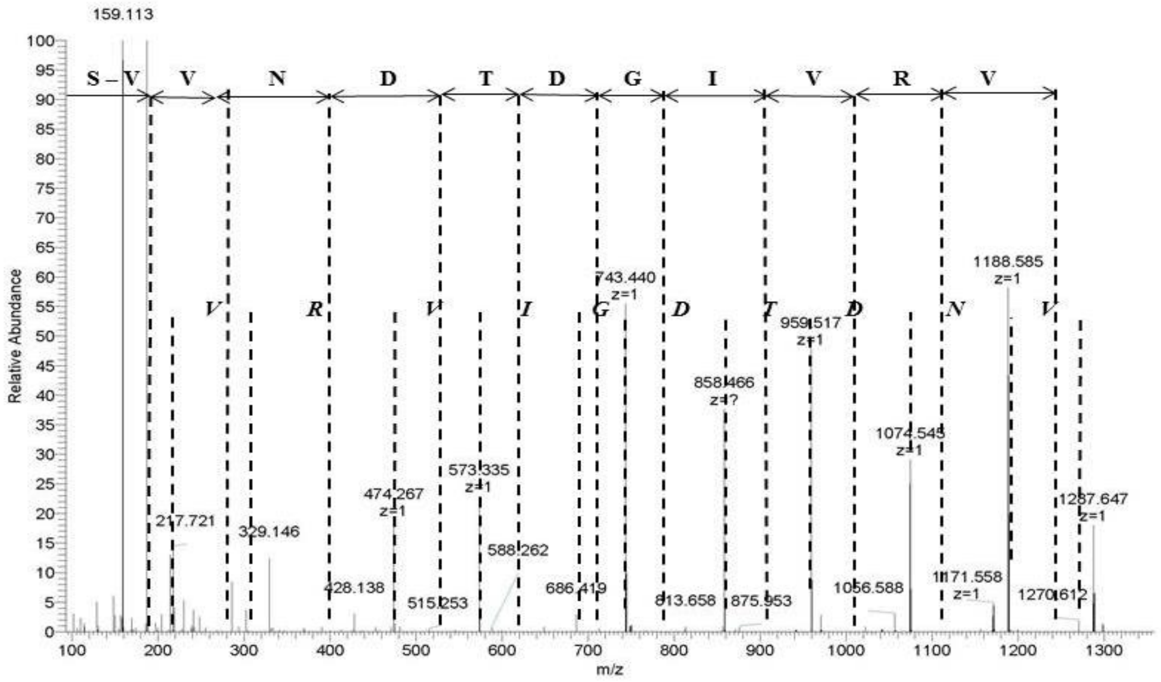

(C)

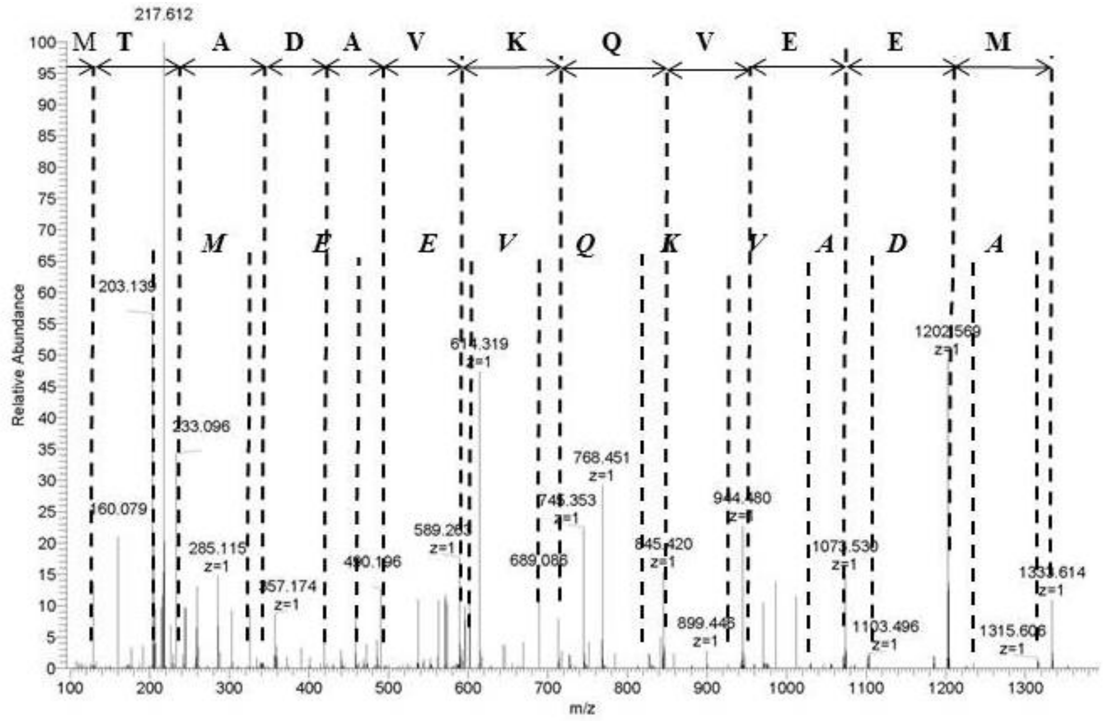

(D)

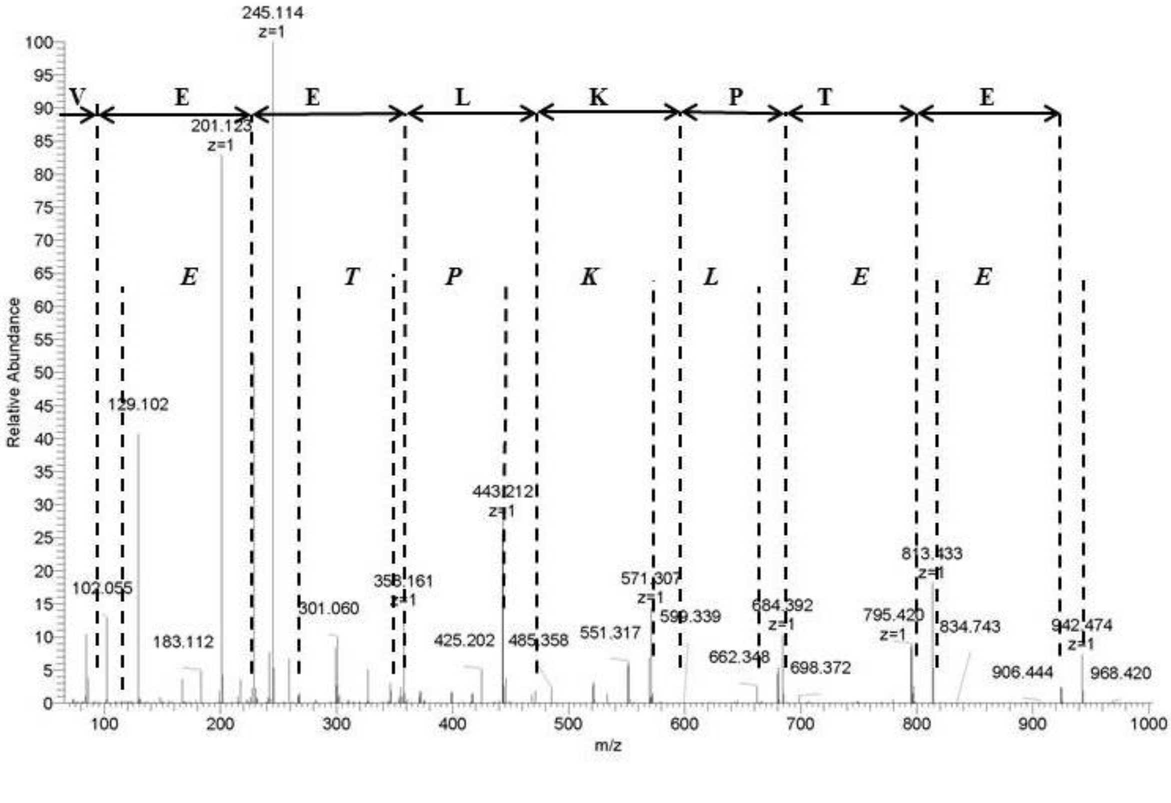

(E)

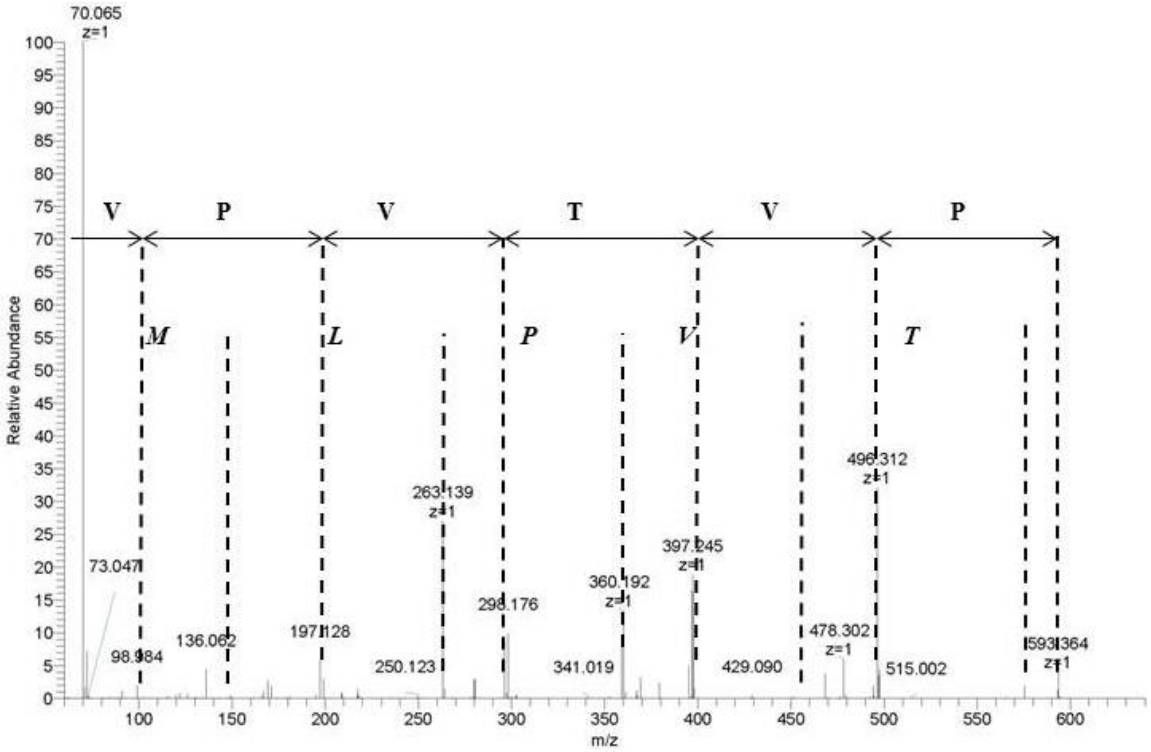
70.065
z=1
V
P
V
T
V
P
M
L
P
V
T
496.312
z=1
263.139
z=1
397.245
z=1
73.047
360.192
z=1
298.176
197.128
136.062
98.984
478.302
z=1
250.123
341.019
429.090
515.002
593.364
z=1
Relative Abundance
m/z

(F)

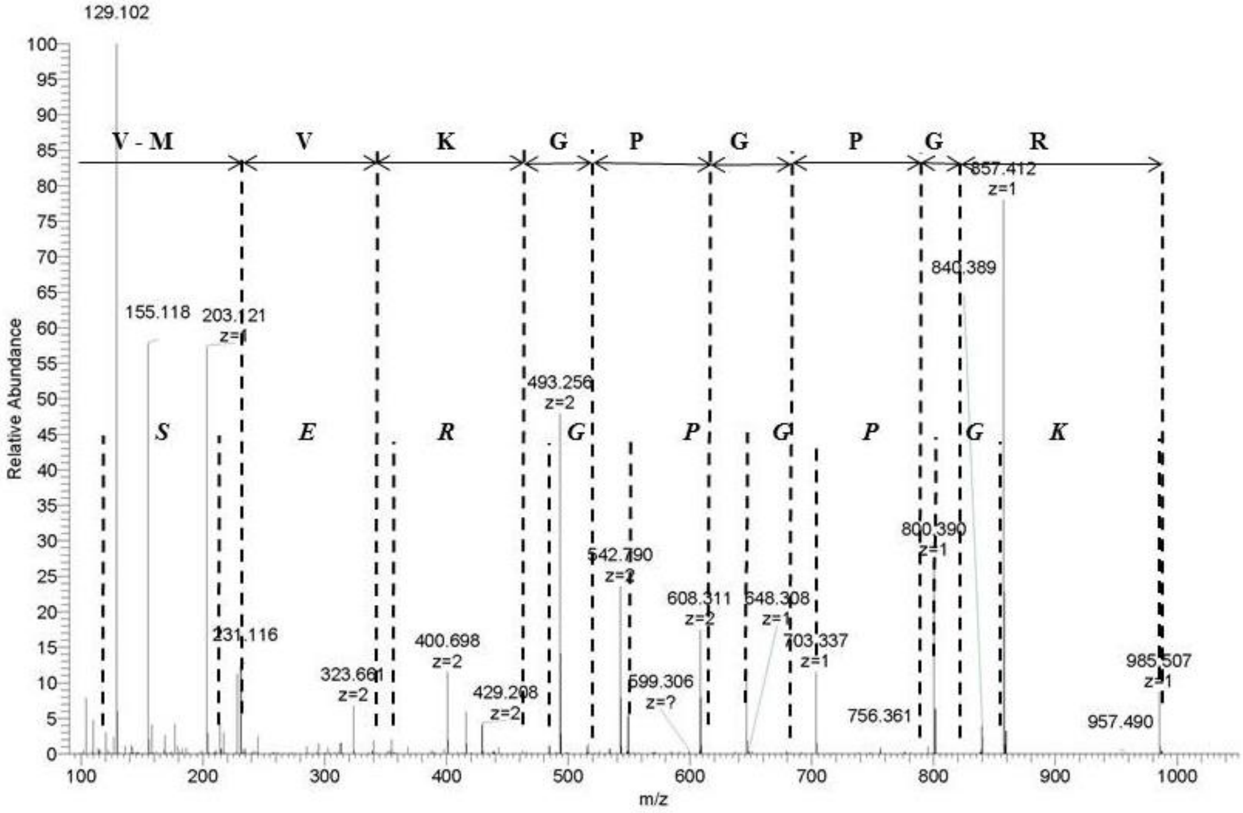
129.102
V - M
V
K
G
P
G
P
G
R
857.412
z=1
840.389
155.118
203.121
z=1
493.256
z=2
S
E
R
G
P
G
P
G
K
800.390
z=1
542.790
z=2
608.311
z=2
648.308
z=1
231.116
703.337
z=1
400.698
z=2
985.507
z=1
323.661
z=2
429.208
z=2
599.306
z=?
756.361
957.490
Relative Abundance
m/z

(G)

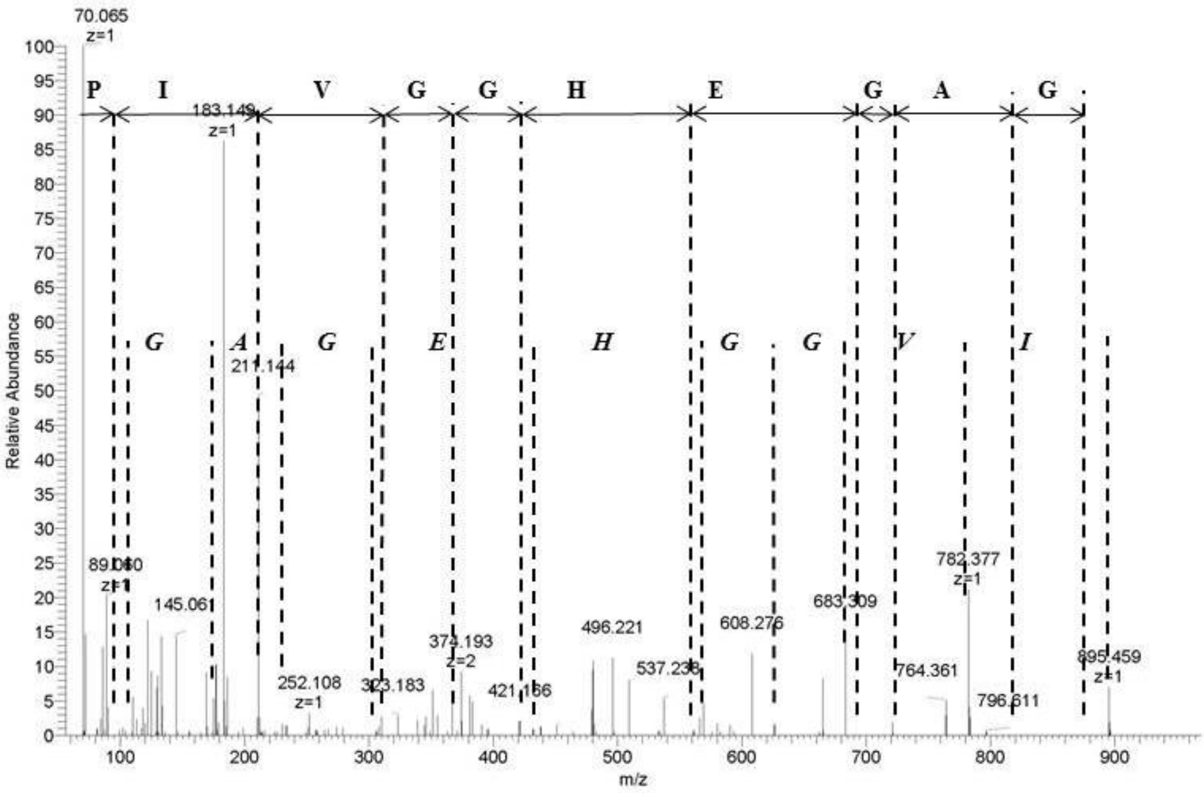
P I V G G H E G A G
G A G E H G G V I
70.065 z=1
183.149 z=1
211.144
89.060 z=1
145.061
252.108 z=1
323.183
374.193 z=2
421.166
496.221
537.238
608.276
683.309
764.361
782.377 z=1
796.611
895.459 z=1
Relative Abundance
m/z

(H)

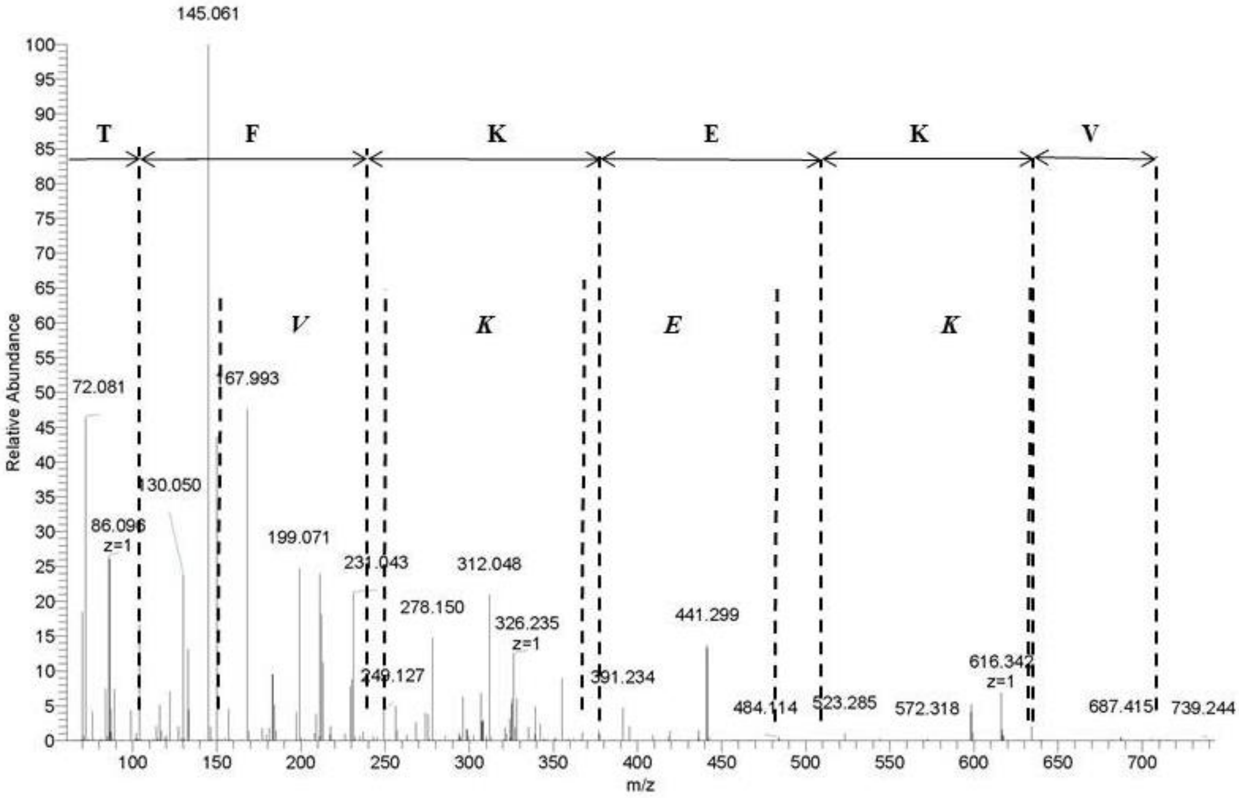
T F K E K V
V K E K
145.061
72.081
167.993
130.050
86.096 z=1
199.071
231.043
312.048
278.150
326.235 z=1
249.127
391.234
441.299
484.114
523.285
572.318
616.342 z=1
687.415
739.244
Relative Abundance
m/z

Figure S2. Manual interpretation of Identified peptide ligands for AmiA **(A)** LQEHSVILIRG **(B)** SVVNDTDGIVRVAE, AliA **(C)** MTADAVKQVEEMLA **(D)** VEELKPTEP **(E)** VPVTVPLM and AliB **(F)** VMVKGPGPGREST **(G)** PIVGGHEGAGV **(H)** TFKEKVM. Manual interpretation of the fragment peaks is shown using non-italic letters for the *b*-ion (fragments appearing to extend from the N-terminal fragment) and italic letters for the *y*-ion.
